# Circulating 4-Hydroxynonenal Is Associated with Clinical Severity in ST-Segment Elevation Myocardial Infarction

**DOI:** 10.3390/ijms27219694

**Published:** 2026-10-29

**Authors:** Ángel Priego-Ranero, Brandon Fisher-Bautista, Rashidi Springall, Luis M. Amezcua-Guerra, Héctor González-Pacheco, Pedro Ruiz-Garcia, Betsabé Jaramillo-Antonio, Malinalli Brianza-Padilla, Gabriela Fonseca-Camarillo

**Affiliations:** 1 Departamento de Inmunología, Instituto Nacional de Cardiología Ignacio Chávez, Mexico City 14080, Mexico; angel.priego@cardiologia.org.mx (Á.P.-R.); bfisherb2400@alumno.ipn.mx (B.F.-B.); maria.springall@cardiologia.org.mx (R.S.); ru419590@uaeh.edu.mx (P.R.-G.); 2 Departamento de Hemodinámica, Instituto Nacional de Cardiología Ignacio Chávez, Mexico City 14080, Mexico; 3 Programa de Maestría en Ciencias Médicas, Universidad Nacional Autónoma de México, Mexico City 04510, Mexico; 4 Unidad de Cuidados Coronarios, Instituto Nacional de Cardiología Ignacio Chávez, Mexico City 14080, Mexico; hector.gonzalez@cardiologia.org.mx; 5 Laboratorio de Medicina Traslacional, Instituto Nacional de Cardiología Ignacio Chávez, Mexico City 14080, Mexico; betsanto@unam.edu

**Keywords:** STEMI, ferroptosis, 4-HNE, GPX4

## Abstract

Oxidative stress and ferroptosis are increasingly recognized as key contributors to the pathogenesis of ST-segment elevation myocardial infarction (STEMI), although the clinical relevance of circulating molecules linked to these processes remains insufficiently defined. The present study evaluated serum levels of the lipid peroxidation marker 4-hydroxynonenal (4-HNE), as well as glutathione peroxidase 4 (GPX4), in patients with STEMI and controls, and explored their relationship with clinical severity. Serum concentrations of 4-HNE and GPX4 were measured, and their associations with clinical risk scores and major outcomes were analyzed using correlation and regression methods. Compared with controls, patients with STEMI showed significantly higher 4-HNE concentrations (median, 1220.2 [IQR, 806–1453] vs. 38.7 [15.6–584]; *p* < 0.001), whereas GPX4 levels were significantly lower in STEMI patients (median, 8.85 [8–10.56] vs. controls 10.50 [9.31–12.11]; *p* = 0.003). Increased 4-HNE levels were positively associated with the TIMI score (ρ = 0.385, FDR-adjusted *p* = 0.026) and Killip–Kimball class (ρ = 0.329, *p* = 0.016; FDR-adjusted *p* = 0.041), suggesting a relationship with greater clinical severity. Although unadjusted analysis indicated an association between higher 4-HNE concentrations and cardiovascular mortality (OR, 1.0033; 95% CI, 1.0004–1.0083; *p* = 0.0176), this finding was not significant after multivariable adjustment. Overall, these findings indicate that STEMI is associated with increased lipid peroxidation and reduced GPX4 levels, supporting the involvement of ferroptosis-related pathways in acute myocardial infarction. Elevated 4-HNE levels were also associated with greater clinical severity.

## 1. Introduction

Cardiovascular diseases (CVDs) remain the leading cause of mortality and disability worldwide, accounting for more than 17.3 million deaths annually, which are largely attributable to acute coronary syndromes (ACSs). According to the Global Registry of Acute Coronary Events (GRACE), ST-segment elevation myocardial infarction (STEMI) represents approximately 36% of ACS cases [1]. STEMI is a life-threatening type of acute myocardial infarction (AMI) characterized by persistent elevation of the ST segment on the electrocardiogram, reflecting ongoing myocardial injury and necrosis [2].

Clinical outcomes following AMI are primarily determined by infarct size, which reflects the extent of cardiomyocyte death. Accumulating evidence has shown that ferroptosis plays a key role in AMI pathogenesis [3].

Ferroptosis is a non-apoptotic regulated cell death driven by iron-dependent lipid peroxidation, resulting in oxidative damage to cellular membranes [4], because of an imbalance between oxidative stress and antioxidant defenses [5]. The process of lipid peroxidation is initiated by reactive free radicals (oxyl, peroxyl, and hydroxyl radicals), which extract electrons from lipids to generate highly reactive intermediates. These intermediates propagate oxidative chain reactions that compromise phospholipid integrity and activate signaling pathways, leading to programmed cell death. Lipid oxidation is initiated through both non-enzymatic reactions, including the Fenton reaction, and enzymatic oxidation catalyzed by lipoxygenases (LOX) [6]. A defining feature of ferroptosis is the progressive accumulation of lipid peroxidation products, including primary lipid hydroperoxides (LOOHs) and secondary reactive aldehydes such as 4-hydroxynonenal (4-HNE) [7]. At low concentrations, these products mediate adaptive redox signaling and metabolic homeostasis by acting as second messengers; however, their accumulation indicates a loss of cellular membrane integrity and advanced signals, and often irreversible, iron-dependent cell death [8].

4-HNE t is one of the most abundant products of lipid peroxidation, playing a key role in the pathogenesis of multiple diseases, including cardiovascular disorders [9], and is a toxic aldehyde generated during the iron-dependent oxidation of polyunsaturated fatty acids (PUFAs). It acts as both a primary driver and a key measurable circulating molecule of ferroptosis [10], and Liu et al. [3] suggest that it could provoke cardiomyocyte death, including cellular apoptosis and necroptosis.

Lipid peroxidation generates a range of reactive aldehydes derived from the oxidative degradation of arachidonic acid and other PUFAs, which can serve as circulating molecules of oxidative stress and mediators of cellular damage [11].

Adducts formed by 4-HNE are involved in numerous cellular processes and may mediate secondary deleterious effects through inducing intra- and intermolecular protein–protein, protein–DNA, and DNA–DNA crosslinking. These covalent modifications can significantly disrupt the biochemical and functional properties of biomolecules, thereby facilitating the onset and progression of diverse pathological conditions [12].

Glutathione peroxidase 4 (GPX4) is a crucial antioxidant enzyme that preserves membrane integrity by preventing lipid peroxidation. Diverse studies have shown that GPX4 expression is negatively associated with endothelial ferroptosis and contributes to the accelerated development of atherosclerosis [13]. In addition to its antioxidant activity, GPX4 regulates iron metabolism by binding iron ions, thereby limiting their participation in the Fenton reaction and reducing oxidative damage [14].

Previous evidence suggests that excessive ROS generation during hypoxia/reoxygenation (H/R) contributes to cardiomyocyte injury through inducing mitophagy, leading to suppression of the ferroptosis-regulating SLC7A11/GSH/GPX4 signaling pathway [15].

Despite these advances, clinical data on ferroptosis-related circulating molecules in patients with STEMI remain scarce. A deeper understanding of these molecular regulators may improve risk stratification and support the development of targeted therapeutic strategies. Therefore, the aim of this study was to determine the circulating levels of 4-HNE and GPX4 in patients with STEMI, and to assess the correlations between these molecules with left ventricular ejection fraction (LVEF), ischemia time, GRACE score, TIMI risk score, and Killip–Kimball class.

## 2. Results

### 2.1. Demographic and Clinical Characteristics

A total of 56 STEMI patients (10 women and 46 men) and 28 controls (14 women and 14 men) were included in this study, and their baseline characteristics and clinical data are shown in Table 1. Their clinical profile and clinical outcomes are also shown in Table 2 and Table 3. Laboratory studies at hospital arrival, including both leukocyte count and differential analysis, as well as inflammatory, endothelial activation, and tissue injury molecules, are shown in Table 4. The angiographic characteristics and outcomes of primary percutaneous coronary intervention are shown in Table 5. The diagram SRTOBE summarizes the number of patients screened, exclusions and their reasons, patients finally included, sample availability and processing, analytes measurements, and availability of clinical and outcome data, outlined in Figure 1.

### 2.2. Concentrations of 4-HNE

Circulating 4-HNE levels were elevated in patients with STEMI compared with control participants (*p* < 0.001). The median 4-HNE concentration was 1220.2 pg/mL (IQR, 806–1453) in the STEMI group and 38.7 pg/mL (IQR, 15.6–584) in the control group, while mean concentrations were 1032 ± 554 and 357 ± 459 pg/mL, respectively (Figure 2).

Additional, 4-HNE was associated with cardiovascular mortality (OR, 1.00033; 95% CI, 1.0004–1.0083; *p* = 0.0176), while no clear association was observed with the remaining variables included in the primary composite outcome of our study. In adjusted models including left ventricular ejection fraction, ischemic time, GRACE score, TIMI score, and Killip–Kimball class (N = 56), the association between 4-HNE concentrations and cardiovascular mortality was attenuated and was no longer statistically significant (OR, 1.0006; 95% CI, 0.9988–1.0078; *p* = 0.322). These findings suggest a potential relationship between circulating 4-HNE and adverse cardiovascular outcomes, although its independent prognostic value remains uncertain.

The 4-HNE serum levels showed positive correlations with clinical risk scores, including TIMI (ρ = 0.385, *p* = 0.005; FDR-adjusted *p* = 0.026) and Killip–Kimball class (ρ = 0.329, *p* = 0.016; FDR-adjusted *p* = 0.041). The association with the GRACE score was weak (ρ = 0.284; *p* = 0.048) and did not remain significant after false discovery rate (FDR) correction (FDR-adjusted *p* = 0.080) (Table 6, Figure 3).

### 2.3. Concentration of GPX4

Circulating GPX4 levels were significantly lower in patients with STEMI than in control participants. The median GPX4 concentration was 8.85 pg/mL (interquartile range [IQR], 8–10.56) in the STEMI group and 10.50 pg/mL (IQR, 9.31–12.11) in the control group. This difference was statistically significant according to the two-sided Mann–Whitney U test (*p* = 0.003). The estimated location shift between groups was 1.39 pg/mL, with a 95% confidence interval of 0.48 to 2.40 pg/mL (Figure 4).

GPX4 levels were not significantly associated with any of the clinical outcomes evaluated. Spearman correlation analysis between GPX4 and clinical parameters showed low-magnitude, non-significant correlation coefficients.

## 3. Discussion

In the present study, we quantified serum levels of the lipid peroxidation product 4-HNE and the antioxidant enzyme GPX4 in patients with STEMI. We observed significantly elevated circulating 4-HNE levels in patients with STEMI compared with controls.

Evidence regarding circulating 4-HNE levels in ACS, particularly during the acute phase of STEMI, remains limited. Previous evidence predominantly originated from experimental models of myocardial ischemia–reperfusion and studies of post-AMI heart failure, with relatively few clinical studies evaluating 4-HNE during the acute ischemic event. Overall, these findings suggest that elevated circulating 4-HNE levels may be associated with a more severe clinical risk profile [16,17,18].

Previous studies in patients with congestive heart failure have shown that circulating 4-HNE levels are elevated and inversely correlated with left ventricular contractility, suggesting the contribution of lipid peroxidation to cardiac dysfunction [18].

In addition, intramitochondrial accumulation of 4-HNE in mice cardiomyocytes following AMI has been associated with mitochondrial dysfunction, ischemic remodeling, and ventricular dysfunction. The authors proposed the monoamine oxidase A (MAO-A)/4-HNE axis as a key mechanism underlying ischemic remodeling and the development of post-STEMI heart failure; however, other ferroptosis-related pathways were not investigated [19].

Furthermore, Sun et al. [20] demonstrated in an in vivo model that increased 4-HNE levels following MI upregulated p53 expression, thereby promoting cardiomyocyte death. They also suggest the cardioprotective role of mitochondrial aldehyde dehydrogenase 2 (ALDH2), which limits 4-HNE accumulation through its detoxification.

Interestingly, we also observed significantly reduced GPX4 levels in patients with STEMI. These findings are consistent with a previous study in which patients with ACS, including unstable angina or AMI, and multivessel coronary artery disease (≥70% stenosis in at least two epicardial coronary arteries) exhibited lower erythrocyte glutathione (GSH) levels than patients with chronic coronary artery disease and healthy controls [21].

In the same study, the ACS group also showed reduced GPX4 activity and increased (malondialdehyde) MDA release from erythrocyte membranes. Similarly, Ceylan et al. [22] reported that GPX and GSH levels were inversely correlated with the SYNTAX and ACEF risk scores, which assess coronary lesion complexity and predict the risk of major adverse cardiovascular events (MACEs), including stroke, myocardial infarction, and cardiovascular death.

Several studies have also suggested a potential therapeutic role for glutathione in STEMI. Patients who received glutathione infusion before primary percutaneous coronary intervention (PCI) exhibited lower H_2_O_2_ levels, reduced neutrophil-to-lymphocyte ratio (NLR), high-sensitivity C-reactive protein (hsCRP), and TNF levels, as well as shorter hospital stays [23,24,25,26].

In addition, ventricular function improved six months after myocardial infarction and PCI in patients treated with glutathione [24]. Although these findings support the beneficial effects of antioxidant therapy, larger randomized clinical studies are needed to establish the efficacy and optimal use of glutathione administration before reperfusion in patients with STEMI.

Evidence regarding GPX activity in ACS remains inconsistent. Several studies have reported reduced GPX activity in patients with ACS [25,26,27,28], and one study also demonstrated an inverse correlation between plasma GPX4 levels and the TIMI risk score, consistent with our findings.

In contrast, Holley et al. [29] reported increased GPX activity, higher mRNA expression, and elevated plasma levels of glutathione peroxidase 3 (GPX3) in patients with ACS compared with those with chronic coronary artery disease and healthy controls. Moreover, the mRNA expression of GPX4 was also increased in ACS.

Notably, GPX4 is the only member of the glutathione peroxidase family capable of reducing complex lipid hydroperoxides, making it the principal endogenous inhibitor of ferroptosis [30]. Consequently, circulating GPX4 levels may have potential value in cardiovascular disease.

Ferroptosis should be considered within the broader inflammatory and oxidative stress. Previous studies have shown that S100A8/A9-associated neutrophil recruitment and complement activation through the lectin pathway contribute to post-ischemic myocardial inflammation, injury, and adverse remodeling [31,32]. These pathways may promote an oxidative environment that can interact with lipid peroxidation and ferroptosis-related mechanisms. However, our findings do not establish a direct causal link between these inflammatory pathways and myocardial ferroptosis, nor do circulating 4-HNE and GPX4 measurements constitute direct evidence of ferroptotic cardiomyocyte death.

The present study has several limitations. First, measurements of circulating molecules do not provide information regarding their cellular or tissue sources and cannot determine which immune or cardiac cell populations may contribute to ferroptosis regulation. Second, the assessment of circulating 4-HNE and GPX4 alone cannot establish the occurrence or extent of ferroptotic cell death in myocardial tissue, and the relatively small sample size and differences in baseline characteristics between groups represent limitations of the study. These findings represent an association between circulating 4-HNE and GPX4 levels and clinical severity in STEMI patients rather than a complete characterization of all the ferroptotic pathways involved.

Future studies with larger and more balanced cohorts will be necessary to confirm and extend these findings.

Mechanistic studies should therefore investigate ferroptosis-related pathways at the cellular level, including differential expression of ferroptosis-regulatory genes in peripheral blood mononuclear cells (PBMCs) and, when feasible, in myocardial tissue.

Nevertheless, our findings support further investigation of ferroptosis-related molecules in ACS, particularly STEMI. The role of ferroptosis in cardiovascular disease remains an emerging field, and future studies should include larger cohorts, longitudinal follow-up, and comprehensive panels of ferroptosis-related molecules and regulatory pathways. Such studies may help determine whether ferroptosis-related markers provide prognostic information beyond established clinical risk factors and may ultimately contribute to the development of individualized therapeutic strategies targeting ferroptosis in patients with cardiovascular disease.

## 4. Materials and Methods

### 4.1. Study Design

A prospective, observational, and analytical study was conducted at the Instituto Nacional de Cardiología Ignacio Chávez in Mexico City.

The study population included patients with ST-elevation myocardial infarction (STEMI), within the first 12 h of symptom onset and (before undergoing primary percutaneous coronary intervention (pPCI)) without prior reperfusion treatment, admitted to the coronary care unit for STEMI according to the Fourth Universal Definition of Myocardial Infarction (2023), from June 2024 to October 2025 [33], and according to the 2023 European Society of Cardiology (ESC) Guidelines for the management of acute coronary syndromes. Patients presented with symptoms suggestive of acute myocardial ischemia lasting ≥20 min and persistent ST-segment elevation in at least two contiguous leads, defined as ST-segment elevation at the J-point of ≥1 mm in all leads except V2–V3, where the thresholds were ≥2.5 mm in men <40 years, ≥2.0 mm in men ≥40 years, and ≥1.5 mm in women. The diagnosis was confirmed by a rise and/or fall in cardiac troponin with at least one value above the 99th-percentile upper reference limit together with clinical and angiographic evidence of acute coronary occlusion. All patients received primary percutaneous coronary intervention (pPCI).

The exclusion criteria for the cases were as follows: patients <40 years and diagnosed with non-ST-elevation MI or unstable angina, patients with type 2 myocardial infarction, autoimmune diseases, and neoplasia disorders.

Control subjects were recruited from the institute staff. The inclusion criteria for the control group were Mexican nationality, age ≥40 years, and no prior history of myocardial infarction. Individuals with a history of acute myocardial infarction, cerebrovascular events, cardiovascular surgery, cardiomyopathies, autoimmune diseases, neoplasia disorders, or immunosuppressive conditions were excluded from the control group. Before enrollment, all control participants received detailed information about the study and provided written informed consent.

Written informed consent was obtained from all participants (cases and controls) or their legal representatives, authorizing the use of blood samples and associated clinical data for research purposes. The study protocol was reviewed and approved by the local Ethics Committee (protocol No. 24-1446), and all study procedures were carried out in compliance with the ethical principles of the Declaration of Helsinki, its subsequent amendments, and applicable local regulations.

The primary endpoint of the study was the comparison of serum concentrations of 4-HNE and GPX4 between patients with STEMI and the control group.

The secondary endpoints were the evaluation of the associations between serum 4-HNE and GPX4 concentrations and clinical severity and relevant clinical characteristics among patients with STEMI, including their relationship with the clinical risk scores and clinical outcomes evaluated in the study.

### 4.2. Clinical Assessment

Demographic, clinical, and laboratory information was collected from each patient upon hospital admission. Demographic characteristics, cardiovascular risk factors, clinical presentation, and relevant laboratory parameters were recorded at hospital admission. Clinical risk and short-term prognosis were assessed upon admission to the Coronary Care Unit using the Global Registry of Acute Coronary Events (GRACE) and Thrombolysis in Myocardial Infarction (TIMI) risk scoring systems. In addition, hemodynamic severity was classified according to the Killip–Kimball system [34,35].

The associations of circulating GPX4 and 4-HNE concentrations with cardiovascular death, cerebrovascular events, and post-infarction ventricular dysfunction were evaluated using logistic regression models. Post-infarction left ventricular dysfunction was defined as a left ventricular ejection fraction (LVEF) ≤ 40%, documented by transthoracic echocardiography after coronary reperfusion and before hospital discharge. LVEF was preferably quantified using the biplane Simpson method from apical two- and four-chamber views. When more than one echocardiographic examination was available, the assessment performed closest to hospital discharge was used [34]. A composite clinical outcome was additionally defined as the occurrence of at least one of these events during follow-up, including cardiovascular death, cerebrovascular events, or post-infarction ventricular dysfunction. Each circulating molecule was analyzed separately as a continuous variable.

### 4.3. Serum Level Determination

A sample of venous blood (4 mL) was obtained from each subject (before undergoing primary percutaneous coronary intervention (pPCI)), collected into tubes without anticoagulant, and was allowed to clot before centrifugation. They were subsequently centrifuged for 15 min at 1507× *g* and the resulting serum was immediately separated and stored at −70 °C until analysis.

4-HNE and GPX4 levels were measured in the respective serum samples of the study participants by using a commercial ELISA kit following the manufacturer’s instructions. 4-HNE was measured by an enzyme-linked immunosorbent assay following the manufacturer’s protocol (Catalog No: EU0187, FineTest, [Wuhan Fine Biotech Co., Ltd., Wuhan, China]; range [31.25–2000 pg/mL]). Serum 4-HNE concentrations were measured using a commercial ELISA kit (FineTest, catalog No. EU0187; manufacturer’s stated measurement range: 31.25–2000 pg/mL), according to the manufacturer’s instructions. Concentrations were calculated by interpolation from the standard curve generated in each assay. Numerical values below the lower limit of the manufacturer’s stated measurement range were retained as obtained, without substitution or imputation, and included in the statistical analyses. These measurements were interpreted cautiously because their analytical performance below the stated measurement range was not independently validated. GPX4 was measured by an enzyme-linked immunosorbent assay following the manufacturer’s protocol (Catalogue No: EEL155; Thermo Fisher Scientific Inc., Vienna, Austria; range: [1.8–23.2 pg/mL]). The absorbance at 450 nm was recorded using a microplate reader.

### 4.4. Statistical Analysis

Clinical, biochemical, and angiographic characteristics were compared between patients with ST-segment elevation myocardial infarction (STEMI) and control participants. Continuous variables are presented as the mean ± standard deviation (SD) or median (interquartile range [IQR]), according to their distribution, whereas categorical variables are expressed as frequencies and percentages. Between-group differences were assessed using Student’s t-test or the Mann–Whitney U test for continuous variables and the chi-square test or Fisher’s exact test for categorical variables, as appropriate.

Serum levels of ferroptosis-related molecules were compared between groups using the appropriate statistical tests according to data distribution, with nonparametric methods applied to circulating molecules exhibiting skewed distributions. To account for multiple comparisons in the circulating molecule and correlation analyses, *p*-values were adjusted using the Benjamini–Hochberg false discovery rate (FDR) procedure.

Associations between circulating biomarker concentrations and indicators of clinical severity, including left ventricular ejection fraction, ischemia time, GRACE score, TIMI risk score, and Killip–Kimball class, were evaluated using Spearman’s rank correlation coefficient. Given the limited number of clinical outcome events, associations between circulating 4-HNE concentrations and adverse clinical outcomes were explored using Firth’s bias-reduced penalized logistic regression to minimize small-sample bias and address potential separation. Odds ratios (ORs) and corresponding 95% confidence intervals (CIs) were estimated using profile penalized likelihood, whereas *p*-values were obtained from penalized likelihood ratio tests. Unadjusted models were fitted for individual clinical outcomes, followed by an exploratory multivariable analysis of cardiovascular mortality. Given the low number of events relative to the number of model parameters, adjusted estimates were interpreted cautiously because of the potential for overfitting. The Benjamini–Hochberg false discovery rate (FDR) correction was applied to exploratory correlation analyses, whereas nominal *p*-values were reported for clinical outcome models without multiplicity adjustment. All statistical tests were two-sided. Statistical findings were interpreted considering effect sizes, 95% CIs, and the exploratory nature of the analyses, with FDR-adjusted *p*-values used to evaluate the correlation analyses. Statistical analyses were performed using R software (R Core Team, 2026, version 2026.04.0).

## 5. Conclusions

In conclusion, our findings demonstrate an association between circulating 4-HNE levels and clinical severity in patients with STEMI, supporting the potential involvement of ferroptosis-related processes in the pathophysiology of acute myocardial infarction. However, circulating 4-HNE and GPX4 levels should not be interpreted as direct evidence of myocardial ferroptosis. Further prospective and mechanistic studies are warranted to determine the prognostic significance of circulating 4-HNE and to establish whether modulation of ferroptosis-related pathways may have therapeutic relevance in acute myocardial infarction.

## Figures and Tables

**Figure 1:**
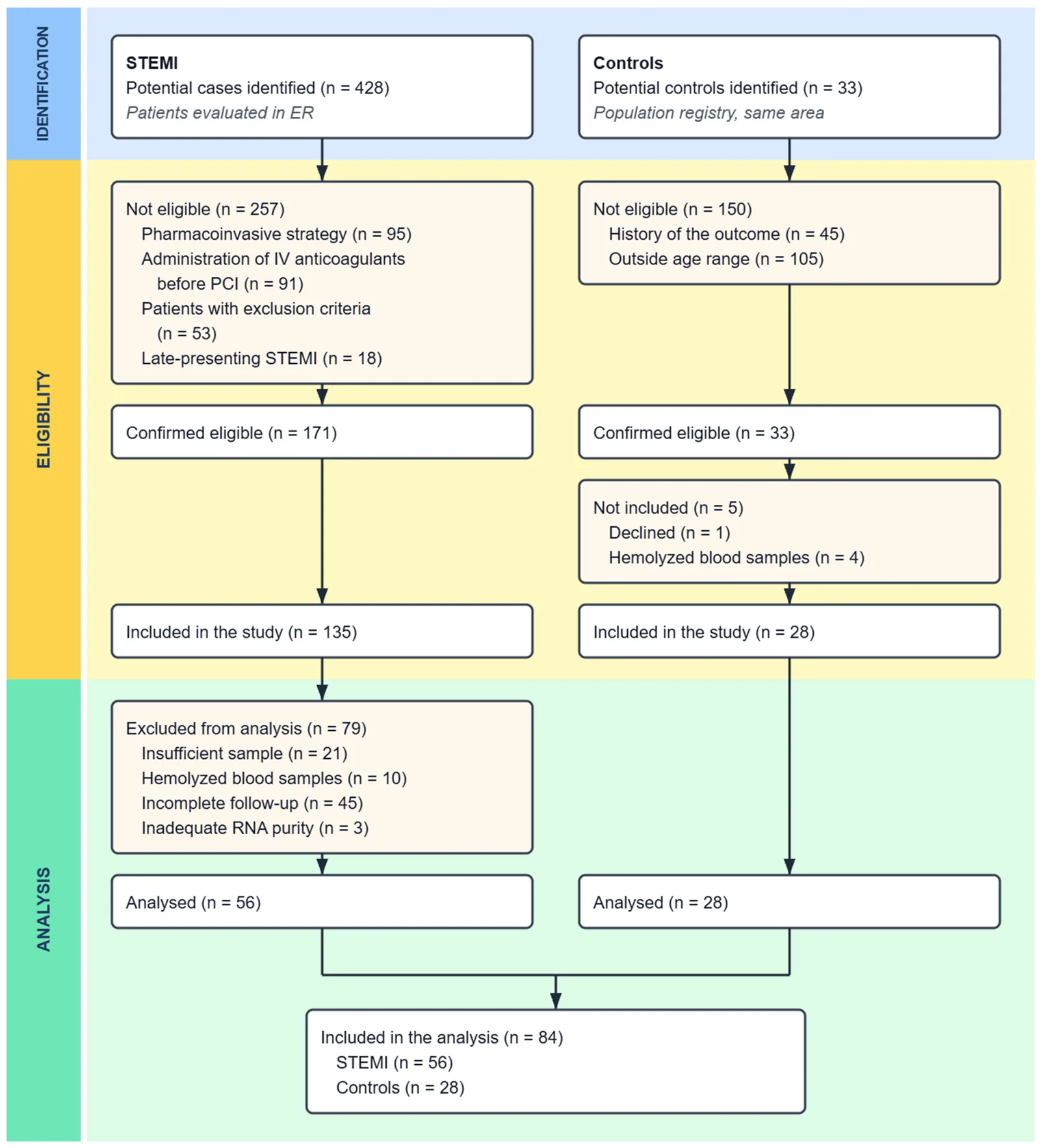
STROBE flow diagram of participant selection in the case–control study. Potential STEMI cases and controls were identified and assessed for eligibility according to predefined inclusion and exclusion criteria. After exclusions at the eligibility and analysis stages, 56 patients with STEMI and 28 controls were included in the final analysis.

**Figure 2:**
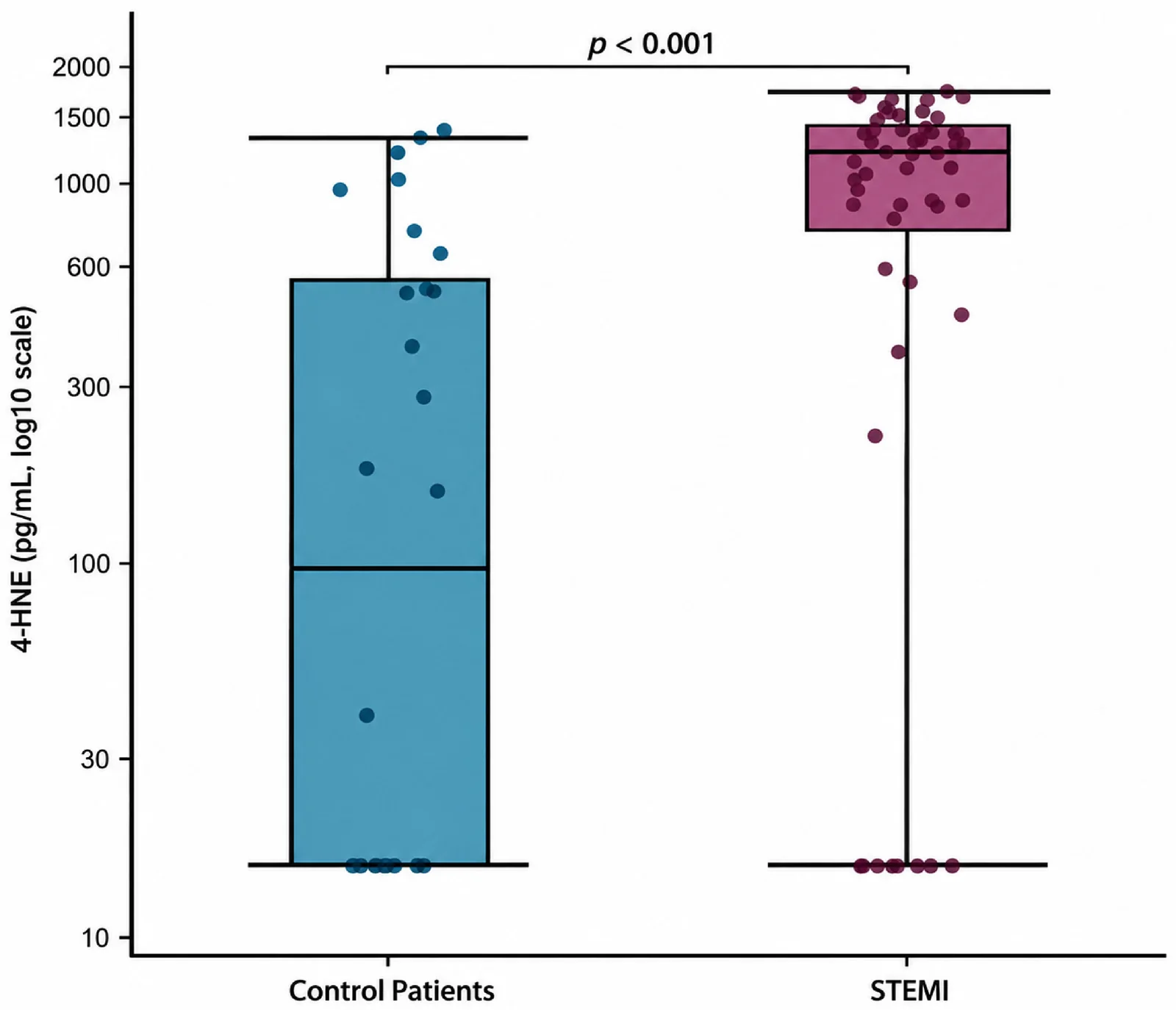
Circulating 4-hydroxynonenal (4-HNE) levels in patients with ST-segment elevation myocardial infarction (STEMI) and control participants. Boxplots illustrate the distribution of serum 4-HNE concentrations in both groups. The central horizontal line represents the median, while the boxes indicate the interquartile range (IQR; 25th–75th percentiles). Whiskers extend to the most extreme observed values within 1.5 × IQR of the lower and upper quartiles, and individual dots represent measurements from each study participant, including observations beyond the whisker limits. Concentrations are displayed on a logarithmic (log10) scale to accommodate the wide distribution of values. Median 4-HNE concentrations were 1220.2 pg/mL (IQR: 806–1453) in the STEMI group and 38.7 pg/mL (IQR: 15.6–584) in the control group. Between-group differences were assessed using the two-sided Mann–Whitney U test (*p* < 0.001). Abbreviations: 4-HNE, 4-hydroxynonenal; STEMI, ST-segment elevation myocardial infarction; IQR, interquartile range.

**Figure 3:**
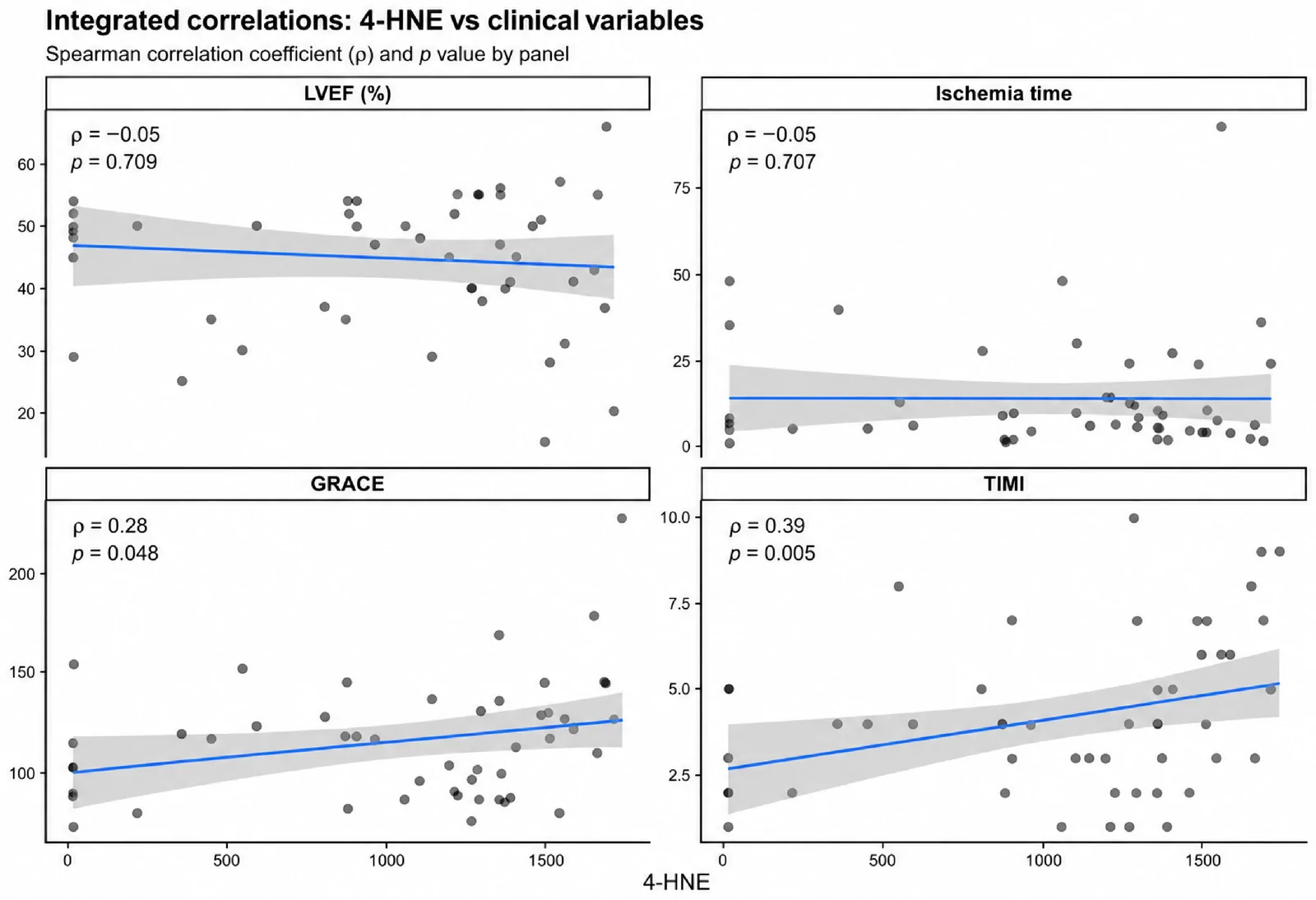
**Integrated correlations between 4-hydroxynonenal (4-HNE) and clinical severity variables.** Scatterplots show the association of 4-HNE concentrations with left ventricular ejection fraction (LVEF), ischemia time, GRACE score, and TIMI score. Spearman’s rank correlation coefficient (ρ), *p* value, and sample size for each comparison are displayed within the corresponding panel. Significant positive correlations were identified for GRACE and TIMI scores, while no significant associations were observed for LVEF or ischemia time. Blue lines denote fitted linear trends and gray bands indicate the 95% confidence interval.

**Figure 4:**
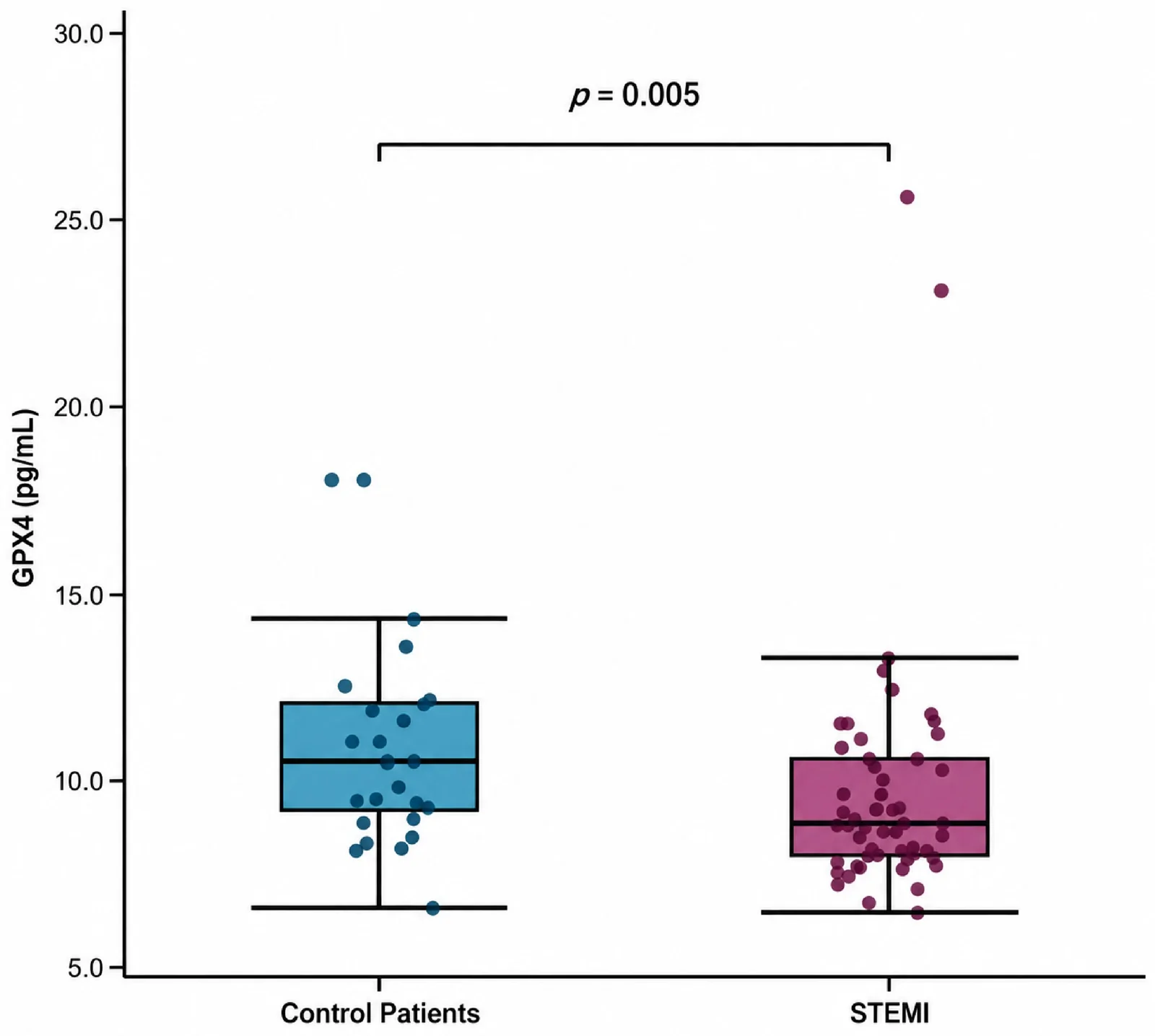
Circulating glutathione peroxidase 4 levels in patients with ST-segment elevation myocardial infarction and controls. Boxplots show the median and interquartile range, whiskers extend to the most extreme observations within 1.5 times the interquartile range, and individual dots represent study participants. Median GPX4 levels were 10.50 pg/mL (IQR, 9.31–12.11) in control participants and 8.85 pg/mL (IQR, 8–10.56) in patients with STEMI. Groups were compared using a two-sided Mann–Whitney U test (*p* = 0.003). Extreme high values were omitted from the displayed *y*-axis range to improve visualization, but all observations were retained in the statistical analysis. GPX4, glutathione peroxidase 4; STEMI, ST-segment elevation myocardial infarction.

**Table 1:** Baseline demographic characteristics, cardiovascular risk factors, comorbidities, and previous medical treatment.

Characteristic	Control N = 28	STEMI N = 56	*p* Value
Age, years	56.0 [51.0, 63.5]	61.0 [55.0, 69.0]	0.088
Sex			0.004
Female	14 (50.0%)	10 (17.9%)	
Male	14 (50.0%)	46 (82.1%)	
Body mass index, kg/m^2^	28.7 [26.1, 31.3]	27.6 [25.4, 31.2]	0.230
Current or previous smoking	6 (21.4%)	32 (57.1%)	0.002
Alcohol consumption	3 (10.7%)	14 (25.0%)	0.157
Illicit drug use	0 (0.0%)	4 (7.1%)	0.296
Hypertension	3 (10.7%)	28 (50.0%)	<0.001
Diabetes mellitus	6 (21.4%)	21 (37.5%)	0.215
Dyslipidemia	5 (17.9%)	9 (16.1%)	>0.999
Chronic kidney disease	0 (0.0%)	3 (5.4%)	0.547
Acetylsalicylic acid	0 (0.0%)	2 (3.6%)	
P2Y12 inhibitor	0 (0.0%)	4 (7.1%)	
Statins	0 (0.0%)	3 (5.4%)	
Fibrates or ezetimibe	0 (0.0%)	1 (1.8%)	
Oral anticoagulants	0 (0.0%)	1 (1.8%)	
Antihypertensive treatment	0 (0.0%)	23 (41.1%)	
Spironolactone	0 (0.0%)	3 (5.4%)	
Furosemide or other diuretics	0 (0.0%)	4 (7.1%)	
Oral antidiabetic agents	0 (0.0%)	11 (19.6%)	
Insulin	0 (0.0%)	2 (3.6%)	

**Table 2:** Clinical profile of patients presenting with ST-segment elevation myocardial infarction.

Clinical Characteristic	STEMI Patients N = 56 ^1^
GRACE risk score	115.3 ± 27.7
TIMI risk score	4.0 ± 2.1
Killip–Kimball class	
I	35 (62.5%)
II	15 (26.8%)
III	5 (8.9%)
IV	1 (1.8%)
Total ischemic time, h	6.5 [4.0, 13.0]
Length of hospital stay, days	4.0 [2.0, 6.0]
Left ventricular ejection fraction, %	43.4 ± 12.5
Vasoactive drug requirement	2 (3.6%)

*^1^ Mean ± SD; n (%); Median [Q1, Q3]. Data are presented as mean ± standard deviation, median [interquartile range], or number (percentage), as appropriate. GRACE, Global Registry of Acute Coronary Events; TIMI, Thrombolysis in Myocardial Infarction.*

**Table 3:** Clinical outcomes among patients with ST-segment elevation myocardial infarction.

Clinical Outcome	STEMI Patients N = 56 ^1^
Cardiovascular death	6 (10.7%)
Left ventricular dysfunction	16 (28.5%)
Stroke	1 (1.8%)
Composite adverse clinical outcome ^2^	19 (33.9%)

*^1^ n (%). ^2^ Composite adverse clinical outcome was defined as the occurrence of at least one of the following: cardiovascular death, left ventricular dysfunction, or stroke. Data are presented as number (percentage). STEMI, ST-segment elevation myocardial infarction.*

**Table 4:** Laboratory characteristics and ferroptosis-related molecules according to the study group.

Laboratory Variable	Control N = 28 ^1^	STEMI N = 56 ^1^
4-Hydroxynonenal, pg/mL	38.7 [15.6–584]	1220.2 [806–1453]
Glutathione peroxidase 4	10.50 [9.31–12.11]	8.85 [8–10.56]
Leukocyte count	-	10.82 [9.13–14.23]
Neutrophil count	-	8.14 [6.36–11.80]
Lymphocyte count	-	1.74 [1.26–2.36]
Neutrophil-to-lymphocyte Ratio	-	5.04 [3.04–7.82]
Platelet count	-	259.5 [221.0, 307.5]
Platelet-to-lymphocyte ratio	-	140.92 [91.13, 207.92]
Serum glucose, mg/dL	-	136.5 [119.0–206.0]
Total cholesterol, mg/dL	-	168.5 [119.5–207.5]
LDL cholesterol, mg/dL	-	102.0 [64.2–135.5]
HDL cholesterol, mg/dL	-	39.9 [33.1–47.4]
Triglycerides, mg/dL	-	117.5 [83.9–191.0]
Ferritin	-	243.5 [102.0–399.0]
Serum iron	-	65.3 [48.0–104.0]
Blood urea nitrogen, mg/dL	-	16.9 [13.2–22.9]
Serum creatinine, mg/dL	-	1.04 [0.84–1.25]
Creatine kinase-MB	-	40.9 [4.7–184.5]
Cardiac troponin T	-	1104.00 [111.00–3402.00]
N-terminal pro-B-type natriuretic peptide	-	841.0 [186.5–2976.5]
C-reactive protein	-	6.12 [2.50–45.50]
Serum lactate	-	2.10 [1.75–2.90]

*^1^ n (%); Mean ± SD; Median [Q1, Q3]. Data are presented as mean ± standard deviation, median [interquartile range], or number (percentage), as appropriate. TIMI, Thrombolysis in Myocardial Infarction.*

**Table 5:** Angiographic and procedural characteristics of patients with ST-segment elevation myocardial infarction.

Angiographic or Procedural Characteristic	STEMI Patients N = 56 ^1^
Culprit vessel	
Left anterior descending artery	24 (45.2%)
Right coronary artery	23 (41.0%)
Left circumflex artery	7 (13.2%)
Left main coronary artery	2 (3.5%)
Initial TIMI flow grade	
0	40 (71.4%)
1	3 (5.4%)
2	8 (14.3%)
3	5 (8.9%)
Final TIMI flow grade	
0	2 (3.5%)
1	1 (1.7%)
2	8 (15.09%)
3	45 (80.3%)
Contrast volume, mL	148.9 ± 76.5
Fluoroscopy time, min	15.5 [10.0, 20.5]

*^1^ n (%); Mean ± SD; Median [Q1, Q3]. Data are presented as mean ± standard deviation, median [interquartile range], or number (percentage), as appropriate. TIMI, Thrombolysis in Myocardial Infarction.*

**Table 6:** Correlation Analysis Between 4-HNE and Clinical Variables.

Molecule	Clinical Variable	N = 56	Coefficient Correlation (Spearman ρ)	95% CI	*p* Value	*p*-FDR
**4-HNE**	LVEF		−0.055	−0.331 to 0.230	0.709	0.709
	Ischemia time		−0.054	−0.325 to 0.225	0.707	0.709
	GRACE score		0.284	0.003 to 0.523	0.048	0.080
	TIMI score		0.385	**0.122 to 0.597**	**0.005**	**0.026**
	Killip–Kimball class		0.329	**0.064 to 0.550**	**0.016**	**0.041**

## Data Availability

Research data are available upon reasonable request.

## Abbreviations

The following abbreviations are used in this manuscript:

4-Hydroxynonenal, 4-HNE; Acute Coronary Syndrome, ACS; Acute Myocardial Infarction, AMI; Aldehyde Dehydrogenase 2, ALDH2; Body Mass Index, BMI; blood urea nitrogen, BUN; Cardiovascular Disease, CVDS; chronic kidney disease, CKD; creatine kinase-MB, CK-MB; C-reactive protein, CRP; Global Registry Of Acute Coro-Nary Events, GRACE; Glutathione Peroxidase 1, GPX1; Glutathione Peroxidase 4, GPX4; high-density lipoprotein, HDL; low-density lipoprotein, LDL; High-Sensitivity C-Reactive Protein, HSCRP; Left Ventricular Ejection Fraction, LVEF; Major Adverse Cardiovascular Event, MACE; Neutrophil-To-Lymphocyte Ratio, NLR; N-terminal pro-B-type natriuretic peptide, NT-proBNP; Percutaneous Coronary Intervention, PCI; Polyunsaturated Fatty Acids, PUFAS; platelet-to-lymphocyte ratio, PLR; Reactive Oxygen Species, ROS; ST-Elevation Myocardial Infarction, STEMI; Thrombolysis In Myocardial Infarction, TIMI.

## References

[1] Vogel B., Claessen B.E., Arnold S.V., Chan D., Cohen D.J., Giannitsis E., Gibson C.M., Goto S., Katus H.A., Kerneis M. (2019). ST-segment elevation myocardial infarction. Nat. Rev. Dis. Primers.

[2] Akbar H., Sharma S. (2026). Acute ST-Segment Elevation Myocardial Infarction (STEMI) [Updated 6 October 2024]. StatPearls [Internet].

[3] Liu L., Pang J., Qin D., Li R., Zou D., Chi K., Wu W., Rui H., Yu H., Zhu W. (2023). Deubiquitinase OTUD5 as a Novel Protector against 4-HNE-Triggered Ferroptosis in Myocardial Ischemia/Reperfusion Injury. Adv. Sci..

[4] Jiang X., Stockwell B.R., Conrad M. (2021). Ferroptosis: Mechanisms, biology and role in disease. Nat. Rev. Mol. Cell Biol..

[5] Kuang F., Liu J., Tang D., Kang R. (2020). Oxidative Damage and Antioxidant Defense in Ferroptosis. Front. Cell Dev. Biol..

[6] Liu J., Kang R., Tang D. (2022). Signaling pathways and defense mechanisms of ferroptosis. FEBS J..

[7] Tang D., Chen X., Kang R., Kroemer G. (2021). Ferroptosis: Molecular mechanisms and health implications. Cell Res..

[8] Peleman C., Francque S., Berghe T.V. (2024). Emerging role of ferroptosis in metabolic dysfunction-associated steatotic liver disease: Revisiting hepatic lipid peroxidation. EBioMedicine.

[9] Santin Y., Fazal L., Sainte-Marie Y., Sicard P., Maggiorani D., Tortosa F., Yücel Y.Y., Teyssedre L., Rouquette J., Marcellin M. (2020). Mitochondrial 4-HNE derived from MAO-A promotes mitoCa2+ overload in chronic postischemic cardiac remodeling. Cell Death Differ..

[10] Requena J.R., Fu M.X., Ahmed M.U., Jenkins A.J., Lyons T.J., Baynes J.W., Thorpe S.R. (1997). Quantification of malondialdehyde and 4-hydroxynonenal adducts to lysine residues in native and oxidized human low-density lipoprotein. Biochem. J..

[11] Pizzimenti S., Ciamporcero E., Daga M., Pettazzoni P., Arcaro A., Cetrangolo G., Minelli R., Dianzani C., Lepore A., Gentile F. (2013). Interaction of aldehydes derived from lipid peroxidation and membrane proteins. Front. Physiol..

[12] Negre-Salvayre A., Coatrieux C., Ingueneau C., Salvayre R. (2008). Advanced lipid peroxidation end products in oxidative damage to proteins. Potential role in diseases and therapeutic prospects for the inhibitors. Br. J. Pharmacol..

[13] Lv Y., Zhang S., Weng X., Huang J., Zhao H., Dai X., Bai X., Bao X., Zhao C., Zeng M. (2023). Estrogen deficiency accelerates postmenopausal atherosclerosis by inducing endothelial cell ferroptosis through inhibiting NRF2/GPX4 pathway. FASEB J. Off. Publ. Fed. Am. Soc. Exp. Biol..

[14] Xu C., Sun S., Johnson T., Qi R., Zhang S., Zhang J., Yang K. (2021). The glutathione peroxidase Gpx4 prevents lipid peroxidation and ferroptosis to sustain Treg cell activation and suppression of antitumor immunity. Cell Rep..

[15] Chen B., Fan P., Song X., Duan M. (2024). The role and possible mechanism of the ferroptosis-related SLC7A11/GSH/GPX4 pathway in myocardial ischemia-reperfusion injury. BMC Cardiovasc. Disord..

[16] El-Mahdy R.I., Mostafa M.M., EL-Deen H.S. (2019). Serum Zinc Measurement, Total Antioxidant Capacity, and Lipid Peroxide Among Acute Coronary Syndrome Patients With and Without ST Elevation. Appl. Biochem. Biotechnol..

[17] Mali V.R., Palaniyandi S.S. (2014). Regulation and therapeutic strategies of 4-hydroxy-2-nonenal metabolism in heart disease. Free Radic. Res..

[18] Csala M., Kardon T., Legeza B., Lizák B., Mandl J., Margittai É., Puskás F., Száraz P., Szelényi P., Bánhegyi G. (2015). On the role of 4-hydroxynonenal in health and disease. Biochim. Biophys. Acta.

[19] Breitzig M., Bhimineni C., Lockey R., Kolliputi N. (2016). 4-Hydroxy-2-nonenal: A critical target in oxidative stress?. Am. J. Physiol.–Cell Physiol..

[20] Sun A., Zou Y., Wang P., Xu D., Gong H., Wang S., Qin Y., Zhang P., Chen Y., Harada M. (2014). Mitochondrial aldehyde dehydrogenase 2 plays protective roles in heart failure after myocardial infarction via suppression of the cytosolic JNK/p53 pathway in mice. J. Am. Heart Assoc..

[21] Mak S., Lehotay D.C., Yazdanpanah M., Azevedo E.R., Liu P.P., Newton G.E. (2000). Unsaturated aldehydes including 4-hydroxy-2-nonenal are elevated in patients with congestive heart failure. J. Card. Fail..

[22] Ceylan Y., Demir H. (2025). Associations of oxidative stress biomarkers with SYNTAX and ACEF risk scores in acute myocardial infarction: A cross-sectional study. Medicine.

[23] Tanzilli G., Truscelli G., Arrivi A., Carnevale R., Placanica A., Viceconte N., Raparelli V., Mele R., Cammisotto V., Nocella C. (2019). Glutathione infusion before primary percutaneous coronary intervention: A randomised controlled pilot study. BMJ Open.

[24] Tanzilli G., Arrivi A., Placanica A., Viceconte N., Cammisotto V., Nocella C., Barillà F., Torromeo C., Pucci G., Acconcia M.C. (2021). Glutathione Infusion Before and 3 Days After Primary Angioplasty Blunts Ongoing NOX2-Mediated Inflammatory Response. J. Am. Heart Assoc..

[25] Bastani A., Rajabi S., Daliran A., Saadat H., Karimi-Busheri F. (2018). Oxidant and antioxidant status in coronary artery disease. Biomed. Rep..

[26] Arrivi A., Pucci G., Placanica A., Bier N., Sordi M., Dominici M., Carnevale R., Tanzilli G., Mangieri E. (2023). Early and prolonged glutathione infusion favorably impacts length of hospital stay in ST-elevation myocardial infarction patients: A subanalysis of the GSH2014 Trial. Minerva Cardiol. Angiol..

[27] Arrivi A., Barillà F., Carnevale R., Sordi M., Pucci G., Tanzilli G., Prandi F.R., Mangieri E. (2022). Protective Biomolecular Mechanisms of Glutathione Sodium Salt in Ischemia-Reperfusion Injury in Patients with Acute Coronary Syndrome-ST-Elevation Myocardial Infarction. Cells.

[28] Li M.N., Bao B.W., Ding S.Y., Ji C.F., Shi X.J., Gao D.S., Qin G., Wang H.J. (2022). Correlation between plasma glutathione peroxidase 4 and N-acetylneuraminic acid levels with clinical risk stratification and prognosis of patients with acute coronary syndrome. Saudi Med. J..

[29] Holley A., Pitman J., Miller J., Harding S., Larsen P. (2017). Glutathione peroxidase activity and expression levels are significantly increased in acute coronary syndromes. J. Investig. Med. Off. Publ. Am. Fed. Clin. Res..

[30] Weaver K., Skouta R. (2022). The Selenoprotein Glutathione Peroxidase 4: From Molecular Mechanisms to Novel Therapeutic Opportunities. Biomedicines.

[31] Zhong B., King B., Mares R.G., Arbanasi E.M., Yndigegn T., Engelbertsen D., Björkbacka H., Nilsson J., Goncalves I., Blom A. (2026). Activation of the complement lectin pathway is associated with long-term cardiac dysfunction and incident heart failure in acute coronary syndrome patients. Int. J. Cardiol..

[32] Mareş R.G., Sabău A.H., Cocuz I.G., Tomuţ M.E., Szabo I.A., Szőke A.R., Tinca A.C., Jakobsson G., Cotoi O.S., Şchiopu A. (2023). S100A8∕A9 is a valuable biomarker and treatment target to detect and modulate neutrophil involvement in myocardial infarction. Rom. J. Morphol. Embryol. = Rev. Roum. Morphol. Embryol..

[33] Thygesen K., Alpert J.S., Jaffe A.S., Chaitman B.R., Bax J.J., Morrow D.A., White H.D. (2019). ESC Scientific Document Group Fourth universal definition of myocardial infarction (2018). Eur. Heart J..

[34] Fox K.A., Goodman S.G., Klein W., Brieger D., Steg P.G., Dabbous O., Avezum A. (2002). Management of acute coronary syndromes. Variations in practice and outcome; findings from the Global Registry of Acute Coronary Events (GRACE). Eur. Heart J..

[35] Antman E.M., Cohen M., Bernink P.J., McCabe C.H., Horacek T., Papuchis G., Mautner B., Corbalan R., Radley D., Braunwald E. (2000). The TIMI risk score for unstable angina/non-ST elevation MI: A method for prognostication and therapeutic decision making. JAMA.

